# Awareness of Retinal Detachment Symptoms Among Medical Students: A Cross-Sectional Study

**DOI:** 10.7759/cureus.81462

**Published:** 2025-03-30

**Authors:** Abdulaziz Mohammad, Fatimah M Alayed, Lamees Alharbi, Shikhah G Alharbi, Faisal Alkhalifah, Razan S Alfurayji

**Affiliations:** 1 Department of Ophthalmology, College of Medicine, Qassim University, Qassim, SAU; 2 College of Medicine, Qassim University, Qassim, SAU; 3 College of Medicine, Suliman Alrajhi University, Qassim, SAU

**Keywords:** awareness, cross-sectional study, medical education, ophthalmology, retinal detachment, saudi arabia

## Abstract

Introduction

Retinal detachment (RD) is a vision-threatening ocular emergency requiring prompt recognition and intervention to prevent permanent visual impairment. Despite its clinical significance, awareness of RD symptoms among medical students in Saudi Arabia, particularly in the Qassim region, remains unexplored. This study aimed to evaluate the awareness and confidence of medical students in recognizing RD symptoms and identifying educational gaps that could impact early diagnosis and management.

Methods

A cross-sectional study was conducted from September 2023 to July 2024 among 270 medical students from three medical colleges in the Qassim region of Saudi Arabia. Data were collected using a validated self-administered questionnaire covering demographics, academic level, ophthalmology course completion, and RD knowledge. Statistical analysis, including chi-square tests, was performed using SPSS Version 26, with a p-value of <0.05 considered significant.

Results

Among the participants, 70% were female and 87% were aged 20-25 years. Although 73% had completed an ophthalmology course, only 7.8% had encountered RD cases. The most recognized symptom was flashing lights (25.1%), followed by floaters (22.2%). Misconceptions were prevalent, with 21.1% believing that RD was always associated with pain and 6.7% perceiving it as self-limiting. Confidence in identifying RD symptoms was low, with only 4.4% of the students feeling very confident. Significant associations were found between academic level and knowledge of RD symptoms (p=0.01) and perceptions of its self-limiting nature (p=0.02).

Conclusion

This study highlights substantial gaps in medical students' awareness and confidence in identifying RD symptoms and emphasizes the need for targeted educational interventions. Enhancing ophthalmological training in medical curricula could improve early detection and management, ultimately contributing to improved patient outcome.

## Introduction

Retinal detachment (RD) is a serious ocular condition characterized by the separation of the neurosensory retina from the underlying retinal pigment epithelium, which can lead to permanent vision loss if not promptly treated. This condition is primarily classified into three types: rhegmatogenous, tractional, and exudative. In some cases, a combination of these types may occur [1]. Rhegmatogenous RD is the most common type, resulting from full-thickness retinal tears that allow liquefied vitreous to enter the subretinal space, leading to detachment [2]. The pathophysiology of RD involves several mechanisms. Retinal breaks are critical in rhegmatogenous cases and vitreous liquefaction plays a significant role in facilitating the detachment process. Tractional RD, on the other hand, is often associated with conditions such as proliferative diabetic retinopathy, in which fibrous tissue exerts traction on the retina, causing detachment. Exudative RD occurs due to the accumulation of fluid beneath the retina without any breaks and is often seen in inflammatory or neoplastic conditions [1]. Prompt diagnosis and treatment are essential to minimize the morbidity associated with RD. In the general population, the prevalence of RD is approximately 0.08% but can increase to 0.7% or higher following cataract surgery [3]. Posterior capsular rupture is a significant risk factor during cataract surgery. Additional risk factors include ocular trauma, high myopia, male sex, young age, and diabetes [4].

Research indicates that medical students often lack adequate training and exposure to ophthalmic conditions, including RD, during their education [5]. This knowledge gap can lead to delays in diagnosis and treatment, emphasizing the need for improved educational programs that focus on ocular emergencies [6]. A study in Pakistan assessing the knowledge of myopic medical students regarding RD found that while 96% of participants could accurately define RD, many lacked awareness of its causes, symptoms, and treatment options, with some even holding misconceptions about alternative therapies, such as faith healing and homeopathy. This highlights the need for improved educational efforts to enhance awareness of RD among at-risk populations, including medical students [7]. Similarly, another study conducted by Dadh et al. on 516 medical students at Syrian Private University reported that while overall awareness of emergency eye disorders was reasonable, there were notable differences in knowledge levels. RD was recognized as one of the severe conditions, following glaucoma and occlusive proximal arterial blockage [8]. This underscores the impact of targeted medical education on the understanding of critical ocular conditions. Limited research has been conducted to evaluate the awareness of RD symptoms, and to our knowledge, no studies have specifically examined this awareness among medical students in Saudi Arabia. This study aimed to bridge this gap by assessing awareness of RD symptoms among medical students in the Al-Qassim region. Given the unique demographic and clinical characteristics of the Qassim region, understanding its current level of knowledge is essential. The findings of this study can guide curriculum development and support targeted educational interventions aimed at improving the recognition and management of vision-threatening conditions.

## Materials and methods

This cross-sectional study was conducted from September 2023 to July 2024 among medical students from three medical colleges in the Qassim Region of Saudi Arabia. The colleges included College of Medicine, Main Campus, Qassim University; Sulaiman Al Rajhi University; and Onaizah College of Medicine.

Study population and sampling

This study targeted medical students enrolled in all academic years. A sample size of 270 participants was determined using Raosoft’s online sample size calculator (http://www.raosoft.com/samplesize.html), with a 95% confidence level, 5% margin of error, and an estimated population size of 900 students. The inclusion criteria were enrollment in one of the participating medical colleges and willingness to provide informed consent. Students from other regions or outside the participating institutions were excluded. Participants were selected using convenience sampling, where students voluntarily responded to the online survey invitation.

Data collection instrument

A validated self-administered questionnaire was used to collect data (Appendix). The questionnaire was designed to assess students’ demographic characteristics, academic level, completion of an ophthalmology course, and knowledge and perceptions of RD. Key areas included awareness of RD symptoms, perception of urgency for medical attention, and confidence in identifying RD. A pilot study involving seven students was conducted to evaluate the clarity, relevance, and face validity of the questionnaire. Participants provided feedback on the comprehensibility of questions, response options, and overall structure. Minor modifications were made based on their input to enhance clarity and minimize misinterpretation. These responses were excluded from the final analysis.

Data collection and management

The questionnaire was distributed online via WhatsApp groups associated with participating colleges. Students were informed about the study objectives, and their consent was obtained electronically before completing the survey.

Statistical analysis

Data were analyzed using the Statistical Package for the Social Sciences (SPSS) Version 26 (IBM Corp., Armonk, NY). Descriptive statistics, including frequencies and percentages, were used to summarize demographic characteristics and key findings. Chi-square tests were employed to examine the associations between academic level and RD knowledge or perceptions. Statistical significance was set at p < 0.05.

Ethical considerations

Ethical approval was obtained from the Committee of Research Ethics, Deanship of Scientific Research, Qassim University (Approval number: 24-82-14; 21/03/2024). Participation was voluntary, and informed consent was obtained from all participants. Confidentiality and anonymity were ensured by using coded identifiers and restricting access to the data to the research team.

## Results

A total of 270 medical students participated in this study. Among them, 70% (n=189) were female, and the majority (87%, n=235) were aged between 20 and 25 years. The academic distribution showed that 32.5% (n=88) were in their sixth year, 24.9% (n=67) were in their fifth year, and 14.4% (n=39) were in their fourth year. Notably, 73% (n=197) had completed an ophthalmology course, but only 52.2% (n=141) felt that they had received adequate education about RD. Demographic characteristics are shown in Table 1.

**Table 1:** Demographic characteristics of medical students participating in the study (N = 270)

Variables	Frequency, n (%)
Gender	
Male	81 (30.0%)
Female	189 (70.0%)
Age (years)	
15-20	19 (7.0%)
20-25	235 (87.0%)
25-30	13 (4.8%)
30-35	3 (1.1%)
College of Medicine	
College of Medicine, Main Campus, Qassim University	178 (65.9%)
Onaizah College of Medicine	26 (9.7%)
Sulaiman Al Rajhi University	66 (24.4%)
Year of study	
Internship	20 (7.4%)
First year	3 (1.1%)
Second year	31 (11.5%)
Third year	22 (8.2%)
Fourth year	39 (14.4%)
Fifth year	67 (24.9%)
Sixth year	88 (32.5%)
Have you studied an ophthalmology course yet?	
Yes	197 (73.0%)
No	73 (27.0%)
Do you believe that an ophthalmology course has taught you enough about retinal detachment?	
Yes	141 (52.2%)
No	129 (47.8%)
Have you encountered cases of retinal detachment (during ophthalmology rotation or elective training)?	
Yes	21 (7.8%)
Frequently yes	8 (3.0%)
Occasionally no but I plan to	99 (36.7%)
No and I don't have plans to	142 (52.5%)
Do any individuals you know, such as family or friends, have experienced retinal detachment?	
Yes	40 (14.8%)
No	230 (85.2%)

Knowledge and perceptions of RD symptoms

The most commonly recognized symptom of RD was flashing lights, identified by 25.1% of students, followed by floaters (22.2%, n=60), ocular pain (17.7%, n=48), shadows in peripheral vision (15.5%, n=42), and vision loss (15.1%, n=41). However, misconceptions were prevalent: 21.1% (n=57) of participants believed that RD is always associated with pain, and 6.7% (n=18) perceived it as a self-limiting condition. When asked about trauma as a cause of RD, 17% (n=46) agreed that it is always preceded by trauma, while 54.1% (n=146) disagreed. Furthermore, 68.5% (n=185) correctly identified the elderly as the most vulnerable age group for RD, and 62.2% (n=168) acknowledged that RD may cause progressive vision loss (Table 2).

**Table 2:** Knowledge about retinal detachment symptoms among medical students, including misconceptions and levels of urgency for medical attention.

Variables	Frequency, n (%)
From the following symptoms, select what you believe are associated with retinal detachment	
Flashing lights	68 (25.1%)
Floaters (small strands or clouds that move across your field of vision)	60 (22.2%)
Vision loss (cloudy, irregular, or curtain-like)	41 (15.1%)
Shadow in the peripheral visual field	42 (15.5%)
Ocular pain	48 (17.7%)
Red eye	25 (9.2%)
Lacrimation	7 (2.5%)
Double vision	29 (10.7%)
Eye discharge	15 (5.5%)
Which of the following best describes the relationship between pain and retinal detachment?	
Always associated with pain	57 (21.1%)
Rarely associated with pain	106 (39.3%)
Pain is the main symptom	22 (8.1%)
Pain only in severe cases	85 (31.5%)
Retinal detachment is always preceded by trauma	
Agree	46 (17.0%)
Disagree	146 (54.1%)
I don’t know	78 (28.9%)
Wearing glasses or contact lenses can prevent retinal detachment	
Agree	32 (11.8%)
Disagree	119 (44.1%)
I don’t know	119 (44.1%)
Retinal detachment is a self-limited condition	
Agree	18 (6.7%)
Disagree	184 (68.1%)
I don’t know	68 (25.2%)
What age group is most vulnerable to retinal detachment?	
Children	25 (9.3%)
Young adults	21 (7.8%)
Middle-aged individuals	39 (14.4%)
Elderly	185 (68.5%)
Select the correct statement regarding retinal detachment	
It can only occur in one eye	50 (18.5%)
It always results in complete blindness	45 (16.7%)
It may cause gradual vision loss	168 (62.2%)
It is never a serious condition	7 (2.6%)
How would you rate the urgency of seeking medical attention for suspected retinal detachment symptoms?	
Need to visit ophthalmologist within a week	59 (21.9%)
Not urgent	8 (3.0%)
Very urgent	194 (71.8%)
No need for medical attention	9 (3.3%)
How confident are you in your ability to recognize the signs and symptoms of retinal detachment?	
Very confident	12 (4.4%)
Somewhat confident	61 (22.6%)
Neutral	96 (35.6%)
Not confident	101 (37.4%)

Confidence levels in recognizing RD symptoms

Confidence in recognizing RD symptoms was generally low. Only 4.4% (n=12) of students reported feeling very confident, 22.6% (n=61) were somewhat confident, 35.6% (n=96) were neutral, and 37.4% (n=101) reported no confidence. Confidence levels were observed to increase with academic progression, peaking in the fourth year, but decreased slightly in later years.

Urgency of medical attention for RD symptoms

When assessing the urgency of medical attention, 71.8% (n=194) of students identified RD as requiring immediate care, while 21.9% (n=59) believed that a visit within a week was sufficient. Only 3% (n=8) considered medical attention unnecessary.

Exposure to RD cases

Despite completing an ophthalmology course, only 7.8% (n=21) of students had encountered RD cases during their clinical training. Additionally, 52.5% (n=142) reported having no exposure to RD cases and no plans to seek further experience.

Correlation between academic level and RD knowledge

A chi-square test revealed significant associations between academic level and various aspects of RD knowledge (Table 3).

**Table 3:** Knowledge and perceptions regarding retinal detachment among medical students across different academic years. Significant associations were determined using chi-square tests (p<0.05). *Indicates statistically significant (p<0.05)

Variables	Year of the study							p-Value
	First-year (n=3)	Second year (n=31)	Third year (n=22)	Fourth-year (n=39)	Fifth year (n=67)	Sixth year (n=88)	Intern (n=20)	
Which of the following best describes the relationship between pain and retinal detachment?								
Always associated with pain	0	4	10	13	14	13	3	0.01*
Rarely associated with pain	2	5	4	15	30	40	10	
Pain is the main symptom	0	7	1	3	4	7	0	
Pain only in severe cases	1	15	7	8	19	28	7	
Retinal detachment is always preceded by trauma								
Agree	0	7	7	8	11	13	0	0.13
Disagree	0	8	8	17	45	53	15	
I don’t know	3	16	7	14	11	22	5	
Wearing glasses or contact lenses can prevent retinal detachment								
Agree	0	7	2	8	8	7	0	0.89
Disagree	2	8	7	14	32	42	14	
I don’t know	1	16	13	17	27	39	6	
Retinal detachment is a self-limited condition								
Agree	0	3	3	4	3	5	0	0.02*
Disagree	1	13	6	26	51	70	17	
I don’t know	2	15	13	9	13	13	3	
What age group is most vulnerable to retinal detachment?								
Children	0	5	1	4	4	8	3	0.79
Young adults	0	3	3	1	5	7	2	
Middle-aged individuals	0	5	2	5	11	15	1	
Elderly	3	18	16	29	47	58	14	
Select the correct statement regarding retinal detachment								
It can only occur in one eye	1	7	5	7	13	15	2	0.37
It always results in complete blindness	0	2	5	8	12	17	1	
It may cause gradual vision loss	2	22	11	21	40	55	17	
It is never a serious condition	0	0	1	3	2	1	0	
How would you rate the urgency of seeking medical attention for suspected retinal detachment symptoms?								
Need to visit ophthalmologist within a week	1	9	6	9	12	15	7	0.29
Not urgent	1	1	2	1	0	3	0	
Very urgent	1	21	13	27	52	68	12	
No need for medical attention	0	0	1	2	3	2	1	
How confident are you in your ability to recognize the signs and symptoms of retinal detachment?								
Very confident	1	0	3	1	5	2	1	0.13
Somewhat confident	1	3	0	6	25	21	5	
Neutral	0	10	4	15	21	40	6	
Not confident	2	18	15	17	16	25	8	

The belief that RD is rarely associated with pain showed a significant difference across academic levels (p=0.01), with higher recognition among advanced students, particularly those in their fifth (n=30) and sixth years (n=40). Additionally, the misconception that RD is a self-limited condition decreased significantly with academic progression (p=0.02), as 70 sixth-year students correctly disagreed with this statement compared to only 13 second-year students.

## Discussion

This study sheds light on the awareness and confidence of medical students in the Qassim region regarding RD, a critical ophthalmic emergency. Our findings revealed significant knowledge gaps, particularly in identifying key symptoms and understanding the urgency of seeking medical attention, echoing similar results from studies conducted in other regions [7,8].

The low confidence levels reported by medical students in this study align with prior research emphasizing insufficient exposure to ophthalmic conditions during medical training [5,6]. Although 73% of participants had completed an ophthalmology course, only 7.8% had encountered RD cases, indicating that theoretical education alone is inadequate for fostering clinical competence. The recognition of flashing lights as a symptom by just 25.1% of students underscores the need for targeted educational interventions. Comparable studies have reported variable levels of awareness about RD, with some populations demonstrating better recognition of ocular emergencies such as glaucoma and arterial occlusions over RD [8].

A study involving 351 non-ophthalmologist healthcare practitioners in western Saudi Arabia assessed their knowledge of RD, acute angle-closure glaucoma, temporal arteritis, and central retinal artery occlusion. The results highlighted age-related disparities in awareness; older respondents demonstrated greater knowledge of RD, while younger practitioners were more familiar with temporal arteritis. Notably, the study reported a total knowledge score of 75.21% for RD, underscoring a moderate level of awareness among healthcare providers [9].

Misconceptions persist among medical students, as 21.1% of the participants in our study mistakenly believed that pain is always associated with RD, and 6.7% perceived RD as a self-limiting condition. Additionally, 17.0% of students incorrectly believed that RD is always preceded by trauma. While trauma is indeed a significant cause of RD, its prevalence varies across regions. For instance, trauma has been reported to account for 30% of RD cases in South Africa [10] and 23% in Zaire [11], highlighting the importance of educating students on the diverse etiologies of RD. Such misconceptions could delay diagnosis and worsen patient outcomes.

Interestingly, later-year students displayed better awareness of RD symptoms and urgency. This observation underscores the value of cumulative medical education and clinical exposure in enhancing knowledge. However, even among advanced students, confidence in identifying RD symptoms remained low, indicating that the curriculum needs to emphasize practical, hands-on training. Simulation-based learning and case discussions could be particularly effective in bridging this gap, as suggested by prior research [5]. Globally, RD prevalence varies, but the delay in seeking medical attention is a common challenge. For instance, Okonkwo et al. [12] reported that many patients delayed seeking help by an average of 13.5 months after symptom onset. Such delays highlight the importance of training medical students, who are often the first point of contact in emergency care settings, to recognize RD symptoms promptly.

This study is one of the first studies to evaluate awareness of RD symptoms among medical students in Saudi Arabia. While this study provides valuable insights into medical students' awareness of RD symptoms, several limitations must be acknowledged. First, the reliance on self-reported data via an online questionnaire distributed through WhatsApp may introduce selection bias, as students who were more interested in ophthalmology might have been more likely to participate. This could influence the generalizability of our findings. Another key limitation of this study is that internal reliability testing, such as Cronbach’s alpha, was not performed. Although the questionnaire underwent face and content validation through expert review and a pilot study, the absence of formal statistical validation metrics limits the ability to assess internal consistency. Furthermore, the study sample was highly imbalanced across academic years, with significantly fewer first-year students (n=3) compared to sixth-year students (n=88). This discrepancy may have skewed results, particularly in comparisons between academic levels. Future research should aim for a more balanced distribution to improve the reliability of inter-group comparisons. Additionally, the questionnaire did not explicitly differentiate between "macula-on" and "macula-off" RD cases, which could have led to variability in participant responses regarding the urgency of intervention. Future studies should incorporate more detailed clinical scenarios to assess students' understanding more accurately. Finally, the limited exposure to ophthalmology during medical education may have influenced students’ knowledge of RD. Since ophthalmology is often introduced only in later academic years with limited instructional hours, medical students may not receive adequate training in recognizing ophthalmic emergencies. This highlights the need for curriculum revisions to integrate ophthalmology education earlier and provide more hands-on clinical exposure.

Future studies could employ longitudinal designs to evaluate the impact of targeted educational interventions. The findings of this study call for a revision of ophthalmology training in medical curricula, emphasizing the practical aspects of diagnosing RD and other ocular emergencies. Integrating simulation exercises and real-world clinical scenarios could enhance students' confidence and preparedness. Furthermore, expanding research to other regions in Saudi Arabia would provide a broader understanding of awareness levels nationwide and inform nationwide educational reforms.

## Conclusions

This study highlights significant gaps in the awareness, knowledge, and confidence of medical students in identifying RD, a critical ophthalmic emergency. Despite the majority having completed an ophthalmology course, exposure to RD cases was limited, and misconceptions about its symptoms and urgency persisted. Advanced academic level was associated with better knowledge, but the overall confidence in recognizing RD symptoms remained low. While our findings underscore the importance of educational interventions, it is crucial to avoid implying causal relationships between specific educational strategies and improved RD awareness without longitudinal evidence. Future research should employ longitudinal or interventional study designs to evaluate the direct impact of enhanced ophthalmology training on student competency. To address the identified gaps, we recommend earlier integration of ophthalmology into medical curricula, increased practical exposure through simulation-based learning, and structured case discussions on ophthalmic emergencies. These strategies could provide students with the necessary skills for early detection and referral of RD cases, ultimately improving patient outcomes. Furthermore, we acknowledge the limitations of this study, including imbalanced sample distribution across academic years and potential selection bias due to online data collection. Future studies should aim for a more balanced sampling and multi-institutional collaboration to enhance the generalizability and robustness of findings.

## Appendices

Questionnaire

1.       Gender

·         Male

·         Female

2.       Age

·         15-20

·         20-25

·         25-30

·         30-35

3.       College of Medicine

·         College of Medicine, Main Campus, Qassim University

·         Onaizah College of Medicine

·         Sulaiman Al Rajhi University

4.       Year of study

·         Internship

·         First year

·         Second year

·         Third year

·         Fourth year

·         Fifth year

·         Sixth year

5.       Have you studied an ophthalmology course yet

·         Yes

·         No

6.       Do you believe that an ophthalmology course has taught you enough about retinal detachment

·         Yes

·         No

7.       Have you encountered cases of retinal detachment (during ophthalmology rotation or elective training)

·         Yes

·         Frequently yes

·         Occasionally no, but I plan to

·         No, and I don't have plans to

8.       Do any individuals you know, such as family or friends, have experienced retinal detachment

·         Yes

·         No

9.       From the following symptoms, select what you believe are associated with retinal detachment

·         Flashing lights (correct answer)

·         Floaters (small strands or clouds that move across your field of vision) (correct answer)

·         Vision loss (cloudy, irregular, or curtain-like) (correct answer)

·         Shadow in the peripheral visual field (correct answer)

·         Ocular pain

·         Red eye

·         Lacrimation

·         Double vision

·         Eye discharge

10.   Which of the following best describes the relationship between pain and retinal detachment?

·         Always associated with pain

·         Rarely associated with pain (correct answer)

·         Pain is the main symptom

·         Pain only in severe cases

11.   Retinal detachment is always preceded by trauma

·         Agree

·         Disagree (correct answer)

·         I don’t know

12.   Wearing glasses or contact lenses can prevent retinal detachment

·         Agree

·         Disagree (correct answer)

·         I don’t know

13.   Retinal detachment is a self-limited condition

·         Agree

·         Disagree (Correct answer)

·         I don’t know

14.   What age group is most vulnerable to retinal detachment?

·         Children

·         Young adults

·         Middle-aged individuals (correct answer)

·         Elderly

15.   Select the correct statement regarding retinal detachment

·         It can only occur in one eye

·         It always results in complete blindness

·         It may cause gradual vision loss (correct answer)

·         It is never a serious condition

16.   How would you rate the urgency of seeking medical attention for suspected retinal detachment symptoms?

·         Need to visit ophthalmologist within a week

·         Not urgent

·         Very urgent (correct answer)

·         No need for medical attention

17.   How confident are you in your ability to recognize the signs and symptoms of retinal detachment?

·         Very confident

·         Somewhat confident

·         Neutral

·         Not confident
